# Donor-implanted Guttata in Transplanted Corneal Tissue: Clinical Signs and Impact on Graft Survival

**DOI:** 10.18502/jovr.v20.15749

**Published:** 2025-09-09

**Authors:** Arthur Hammer, Ammatul Takaza, Mark Lane, Azizur Rahman, Alfonso Vasquez-Perez

**Affiliations:** ^1^Moorfields Eye Hospital, NHS Foundation Trust, London, UK; ^2^St George's, University of London, London, UK; ^3^These two authors contributed equally to this work.

**Keywords:** Descemet Membrane Endothelial Keratoplasty, Donor Implanted Guttata, Eye Bank Screening, Fuchs Endothelial Dystrophy, Penetrating Keratoplasty

## Abstract

**Purpose:**

To present our experience with patients who developed donor-implanted guttata (DIG) following uncomplicated corneal endothelial transplantation.

**Case Report:**

Three patients who underwent Descemet membrane endothelial keratoplasty were diagnosed with DIG, which was confirmed by confocal microscopy. Two of the cases received corneas from the same donor and experienced delayed recovery of corneal transparency despite fully attached grafts.

**Conclusion:**

Fuchs endothelial dystrophy is a common condition within the general population and is likely to be present in donor corneas. We assessed three cases of DIG, and the results highlight the need for improved screening of this condition prior to transplantation and its consideration in the early postoperative period if corneal edema is present despite graft attachment.

##  INTRODUCTION

Fuchs endothelial dystrophy (FED) is the most common type of corneal dystrophy. It is characterized by progressive degeneration of endothelial cells, leading to a reduction in corneal deturgescence, which results in edema and a loss of corneal transparency. Clinical features include the appearance of cornea guttata, which corresponds to excrescences in the Descemet's membrane.^[1]^

The prevalence of FED varies across the population. It is likely underdiagnosed due to its asymptomatic nature in its early stages. Prevalence of guttata was reported to be 4% in the US population over 40 years of age.^[2]^

There have been reports of cornea guttata in donor material, and its frequency varies with different studies. Nahum et al found that 4% of donor corneas showed signs of guttata on specular microscopy in early postoperative follow-up.^[3]^ Considering the prevalence of guttata within the general population, this condition may be underdiagnosed in donor corneas due to challenges in identifying early stages of FED during eye bank screening.

We present a case series of patients with donor-implanted guttata (DIG) following Descemet membrane endothelial keratoplasty (DMEK). We discuss the possible pathogenesis of this condition and current methods of eye bank screening, which may be insufficient in identifying guttata in donor tissue prior to transplantation.

##  CASE PRESENTATION

This study received approval from the Institutional Review Board. All patients provided their consent for publication of the article in accordance with ICMJE Recommendations for the Protection of Research Participants.

### Case 1

**Figure 1:**
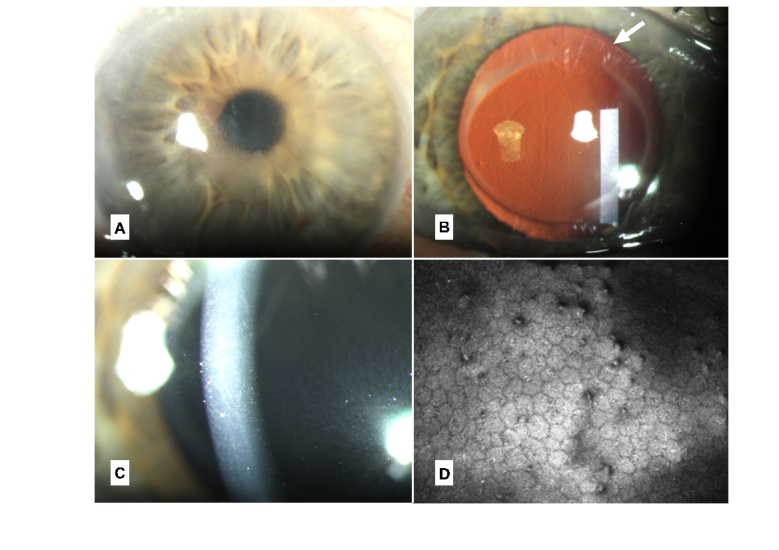
Case 1. (A) Four weeks post DMEK, with central corneal edema despite a fully attached graft. (B) Six weeks post DMEK, central guttata on transillumination. The arrow indicates the border of the graft. (C) Eight weeks post DMEK, corneal edema has resolved, but corneal guttata is present. (D) Confocal image at 8 weeks showing clear guttata in the graft.

A 78-year-old male diagnosed with FED was treated with DMEK surgery combined with simultaneous phacoemulsification and IOL implantation (triple procedure) in both eyes within a 4-month period. Vision improved in his left eye from 20/63 to 20/30 after 1 month; his cornea was clear, and the central corneal thickness (CCT) was 514 µm at month 4. The right eye also underwent uneventful DMEK surgery but was noted to have paracentral residual edema postoperatively, which took 4 weeks to resolve despite a fully attached graft. At the 6-week postoperative visit, confluent guttata (grade 3 in Lorenzetti's classification)^[4]^ was evidenced in the central 3 mm of the cornea in the donor tissue of the right eye, which was confirmed by specular microscopy [Figure 1]. Further review indicated that the donor cornea for the left eye had been reported with 2240 cells/mm^2^. Endothelial cell density at month 6 was 2127 cells/mm^2^ in the right eye and 699 cells/mm^2^ in the left eye. Both corneas remained transparent one year after surgery with best-corrected visual acuity of 20/30.

**Figure 2:**
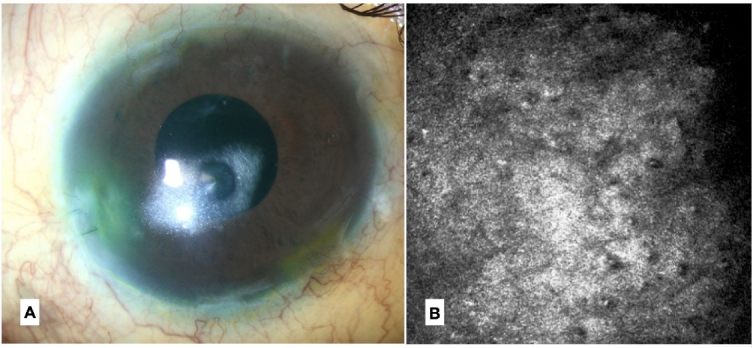
Case 2. This patient received the contralateral cornea from the same donor as in Case 1. (A) Corneal transparency was achieved after 2 months despite consistent graft attachment. (B) Confocal microscopy confirmed that guttata was also present in this graft.

### Case 2 

A 74-year-old male was diagnosed with aphakic bullous keratopathy in his right eye. This eye was amblyopic due to high myopia and had a history of macula-off retinal detachment, which had been treated with pars plana vitrectomy. He was aphakic due to complicated cataract surgery following retinal detachment surgery. DMEK surgery was performed using a safety net technique for aphakia.^[5]^ Postoperatively, persistent corneal edema was present despite full graft attachment, but it resolved after 2 months. Interestingly, the eye bank reported that the tissue for this case was harvested from the contralateral eye of the donor of Case 1. Upon further review, the donor tissue report indicated 2300 cells/mm ${}^{2}$ . Endothelial cell count of the right eye could not be measured with specular microscopy due to corneal scarring, but central guttae were observed on confocal microscopy [Figure 2]. One year after surgery, the eye remained comfortable with a well-functioning graft and counting fingers vision.

### Case 3 

A 90-year-old female with FED underwent uneventful DMEK surgery in her right eye 3 years ago. During the postoperative period, the graft took 2 months to clear, despite being fully attached. The corneal edema eventually cleared, resulting in a best-corrected visual acuity of 20/30. Gradual visual deterioration was noticed at 3 years. Clinical examination indicated corneal edema with central guttata in her DMEK graft (grade 5).^[4]^ Upon further review, the donor tissue report indicated 2420 cells/mm^2^. Her vision dropped to 20/100, and she is now placed on the waiting list for repeat DMEK surgery.

##  DISCUSSION 

In this case series, we present three cases of donor-implanted corneal guttata, and the findings highlight key points linked to this condition. DIG should be considered and actively searched for in patients with persistent corneal edema in the postoperative period, even in the presence of graft attachment, as observed in Cases 1 and 2. Additionally, it should be considered in patients with earlier-than-expected graft failure, as in Case 3. In contrast to the study by Nahum et al, we found that guttata could have negative impacts on both vision and graft survival.^[3]^

A key question is whether this condition can be avoided using current eye bank screening protocols. In the UK, donor endothelial evaluation is performed using light inverted microscopy with cells immersed in a hypotonic solution. Cell number, morphology, and density are analyzed. Slit lamp evaluation is required for each cornea after excision and/or sectioning and prior to implantation. Specular images can be taken, but often only capture a small section of the cornea to provide a cell count. This limited view may not fully disclose the presence of guttae in donor tissue. These tests are operator-dependent, and a thorough assessment may be unreliable in the presence of edematous post-mortem corneas.^[6]^

One method for improving guttata detection would be to use confocal microscopy in pre-transplantation screening, allowing for early detection of guttata, particularly in edematous cadaveric corneas.^[7]^ Unfortunately, confocal microscopy in preoperative screening would have both financial and time implications and would still be subject to operator variability.^[6]^

One way to reduce guttata in donor grafts would be to ensure a rigorous screening process with a specific detection method. Safi et al^[8]^ used inverted light microscopy to gather preoperative endothelial images of donor corneas. Analysis of these images found a positive correlation between postoperative guttata and three semiquantitative criteria: the appearance of blebs (a phrase coined to describe the thickening of the cell membrane), breaks or interruptions within the cell membrane, and images where less than half of the cells were of circular/hexagonal shape. Including these criteria within the current eye bank screening would allow for a more thorough evaluative process and may decrease the prevalence of DIG.

One theory regarding the etiology of guttata would be that Fuchs dystrophy in the donor material is triggered by the recurrence of the host disease. However, we believe guttata due to Fuchs dystrophy seen in grafts are either present in donor tissues during implantation (as seen in Cases 1 and 2) or could develop much later in transplanted corneas from predisposed donors. Most commonly, guttata may have been missed due to limitations in eye bank screening or due to low levels in the donor, which subsequently progress over time, as described in Case 3.^[9]^

In summary, all cases in this study had significant guttata in the donor tissue and were compatible with a diagnosis of FED.^[4]^ DIG due to Fuchs dystrophy is a potentially underdiagnosed condition that may be asymptomatic but can lead to early graft failure, resulting in reduced graft survival. Using an advanced screening system based on inverted light microscopy could help increase the rate of detection prior to transplantation and will be key in reducing the future incidence of this condition.

##  Financial Support and Sponsorship

None.

##  Conflicts of Interest

None.
